# Comprehensive profiling of JMJD3 in gastric cancer and its influence on patient survival

**DOI:** 10.1038/s41598-018-37340-w

**Published:** 2019-01-29

**Authors:** Zhenyu Xu, Yabin Xia, Zhangang Xiao, Yuliang Jia, Lina Li, Yan Jin, Qijie Zhao, Lin Wan, Tao Yi, Yangyang Yu, Qinglian Wen, Yinxin Zhu, Bo Qin, Fan Zhang, Jing Shen

**Affiliations:** 1 Precision Medicine centre, Yijishan Affiliated Hospital of Wannan Medical College, Wuhu, Anhui Province PR China; 2 Department of Gastrointestinal Surgery, Yijishan Affiliated Hospital of Wannan Medical College, Wuhu, Anhui Province PR China; 3 Laboratory of Molecular Pharmacology, Department of Pharmacology, School of Pharmacy, Southwest Medical University, Luzhou, 646000 Sichuan PR China; 4 South Sichuan Institution for Translational Medicine, Luzhou, 646000 Sichuan PR China; 5 Department of Gastroenterology, Yijishan Affiliated Hospital of Wannan Medical College, Wuhu, Anhui Province China; 6 Department of Gynecology and Obstetrics, Yijishan Affiliated Hospital of Wannan Medical College, Wuhu, Anhui Province China; 7 Department of Hematology and Oncology, The Children’s Hospital of Soochow, Jiangsu, China; 8 School of Chinese Medicine, Hong Kong Baptist University, Hong Kong, China; 9 Center for Diabetes, Obesity and Metabolism, Department of Physiology, Shenzhen University Health Science Center, Shenzhen, Guangdong province PR China; 10 Department of Oncology, The Affiliated Hospital of Luzhou Medical College, Luzhou, Sichuan 646000 P.R. China; 11 Department of Gastroenterology, The Third Affiliated Hospital of Soochow University, Changzhou, 213003 Jiangsu PR China; 12 Shenzhen Key Laboratory of Ophthalmology, Shenzhen University, Shenzhen, 518040 Guangdong, China; 13 Department of Pathology, Yijishan Affiliated Hospital of Wannan Medical College, Wuhu, Anhui Province China; 14 Department of Ophthalmology, Shenzhen Shekou People’s Hospital, Shenzhen, Guangdong Province P. R. China

## Abstract

Histone methylation is thought to control the regulation of genetic program and the dysregulation of it has been found to be closely associated with cancer. JMJD3 has been identified as an H3K27 demethylase and its role in cancer development is context specific. The role of JMJD3 in gastric cancer (GC) has not been examined. In this study, JMJD3 expression was determined. The prognostic significance of JMJD3 and its association with clinical parameters were evaluated. JMJD3 dysregulation mechanism and targets were analyzed. The effect of JMJD3 mutation was determined by functional study. Results showed that JMJD3 was overexpressed in different patient cohorts and also by bioinformatics analysis. High JMJD3 expression was correlated with shortened overall survival in patients with GC and was an independent prognosis predictor. Genetic aberration and DNA methylation might be involved in the deregulation of JMJD3 in GC. Downstream network of JMJD3 was analyzed and several novel potential targets were identified. Furthermore, functional study discovered that both demethylase-dependent and demethylase-independent mechanisms were involved in the oncogenic role of JMJD3 in GC. Importantly, histone demethylase inhibitor GSK-J4 could reverse the oncogenic effect of JMJD3 overexpression. In conclusion, our study report the oncogenic role of JMJD3 in GC for the first time. JMJD3 might serve as an important epigenetic therapeutic target and/or prognostic predictor in GC.

## Introduction

Epigenetic modifications play an important role in cancer initiation and progression^1^. Histone methylation is an essential epigenetic phenomenon and the dysregulation of it is associated with the processes of cancer occurrence/progression^2^. The most common histone modifications are acetylation and methylation, which result in target gene expression or repression^3^. The Jumonji domain containing-3 (JMJD3), also known as lysine (K)-specific demethylase 6B (KDM6B) can demethylate H3K27me3 to H3K27me2 or H3K27me1, and dissociate polycomb group complexes^4^.

Many studies have demonstrated that JMJD3 is involved in cancer progression via regulation of several cellular processes, such as proliferation, senescence, and apoptosis^1,3,5^. However, there is controversy regarding the expression pattern of JMJD3 in different cancers. Based on analysis of JMJD3 expression in diverse tumor tissues from the oncomine database, Agger *et al*. found that the expression of JMJD3 was significantly decreased in various cancers, including lung and liver carcinomas, as well as various hematopoietic malignancies^6^. Moreover, JMJD3 expression levels are lower in colorectal cancer relative to normal tissues^7^. Conversely, Shen *et al*. reported that both JMJD3 mRNA and protein levels were significantly elevated in renal carcinoma compared to adjacent normal tissue^8^. Moreover, JMJD3 expression was found to be significantly increased in prostate cancer, and increased further during metastasis^9,10^. Similarly, high expression levels of JMJD3 were found in gliomas^11^.

To our knowledge, there is no published study on the role of JMJD3 in gastric cancer (GC). In the current study, *JMJD3* transcripts and JMJD3 protein expression were measured in different patient cohorts. The clinicalpathological and prognostic significance of JMJD3 expression were evaluated and the upstream regulating mechanism and downstream targets were identified. Elucidation of the role of JMJD3 in GC may lead to new therapeutic approach for the treatment of this disease.

## Materials and Methods

### Gastric clinical tissues

Clinical microarray tissues from 128 gastric cancer patients were retrieved from the tissue bank of the Prince of Wales Hospital (Hong Kong). Use of these tissues had been approved by the Joint Chinese University of Hong Kong—New Territories East Cluster Clinical Research Ethics Committee. A total of 41 fresh gastric cancer and adjacent non-cancerous tumor tissue samples were collected from the tissue bank of Yijishan Hospital of Wannan Medical College (Wuhu, Anhui Province, China). All procedures using human tissue samples were performed in accordance with the relevant guidelines and regulations of the above institutions and informed consent for study participation were obtained from all patients involved.

### RT-PCR and real-time quantitative PCR

Total RNA was extracted from tissues using TRIReagent (Invitrogen, Carlsbad, CA, USA) according to the manufacturer’s protocol. DNase I-treated RNA samples were reverse transcribed using M-MLV reverse transcriptase (Takara) plus a mixture of oligo (dT)12–18 and random primers. cDNA samples (1 µl) were used for conventional PCR amplification, using JMJD3-specific primer pairs. For real-time quantitative PCR analysis, the PCR reaction was performed in a real-time PCR system (Takara) and the expression levels of target gene relative to β-actin were determined using an SYBR Green-based comparative CT method (relative fold change = 2^−ΔΔCT^). Primers used are as follows: JMJD3: forward primer: 5′-GGAGGCCACACGCTGCTAC-3′, reverse primer: 5′-GCCAGTATGAAAGTTCCAGAGCTG-3′, β-actin: forward primer: 5′-CATGTACGTTGCTATCCAGGC-3′, reverse primer: 5′-CTCCTTAATGTCACGCACGAT-3′.

### Immunohistochemistry

Immunohistochemistry of JMJD3 was conducted on a gastric cancer tissue microarray consisting of 128 tumor tissues. Tissue sections were deparaffinized, rehydrated and rinsed in distilled water. Antigen retrieval was done with sodium citrate buffer (pH 6.0), in a microwave oven for 5 min. The endogenous peroxidase activity was blocked using 3% (v/v) hydrogen peroxide for 10 minutes. Immunohistochemical staining for JMJD3 was performed using anti-JMJD3 antibodies (BD Biosciences) via the standard avidin-biotin method. Measurement of immunohistochemical staining was based on a semi quantitative scoring method. For the intensity of staining, 0 = negative (<5%), 1 = very weak (5~20%), 2 = weak (21~40%), 3 = moderate (41~60%), 4 = strong (61~80%), 5 = very strong (>80%). JMJD3 scores in gastric cancer tissues were further subdivided into high-expression (3, 4, 5) and low-expression groups (0, 1, 2).

### Cell lines and transfection

Gastric cancer cell lines TMK-1 and MKN-45 were used in this study. TMK1 was obtained from Dr. Eiichi Tahara (University of Hiroshima, Hiroshima, Japan). MKN-45 was purchased from American Type Culture Collection (Manassas, VA, USA). Transient transfection of pMSCV-wt JMJD3 or pMSCV-mutant JMJD3 was mediated by Lipofectamine 2000.

### MTT assay

Cell viability was measured by 3-(4,5-dimethylthiazol-2-yl)-2,5- diphenylte–trazolium bromide (MTT) assay. After transfection for different time periods, MTT solution at the final concentration of 0.5 mg/ml was added to each well and the plates were incubated for another 3 h. Dimethyl sulfoxide (DMSO) (150 μl) was then added to solubilize MTT tetrazolium crystal. Finally, the optical density was determined at 570 nm.

### BrdU assay

The colorimetric cell proliferation ELISA kit using Bradykinine U (BrdU) incorporation (Roche, Indianapolis, IN, USA) was used for cell proliferation assay. Experiment was carried out according to manufacturer’s instruction.

### Bioinformatics analysis

The relationship of JMJD3 expression and overall survival was determined using TCGA data. The cBioPortal for Cancer Genomics (http://www.cbioportal.org/) provides large scale cancer genomic data and cancer clinical sample information. The association of JMJD3 expression with clinical parameters and DNA methylation, the JMJD3 genetic alteration and downstream targets were analyzed by cBioportal. The comparison of expression level of different JMJD3 transcript variants in GC and normal tissue was carried out in Human Protein Atlas (http://www.proteinatlas.org/). Proteins that were positively co-expressed with JMJD3 and negatively co-expressed with EZH2 were identified by online program Venny using TCGA data.

### Statistical analysis

GraphPad Prism 5 (GraphPad Software) was used for statistical analysis. All results were expressed as mean ± SD from at least three independent experiments. Student t-test was used to compare the discrepancy of two groups and one-way ANOVA was used to compare multiple groups. Overall survival was shown as Kaplan–Meier curve with p values calculated using the log-rank test. P values of less than 0.05 were considered statistically significant.

## Results

### JMJD3 was upregulated in GC and its high expression predicts poor survival

**Figure 1:**
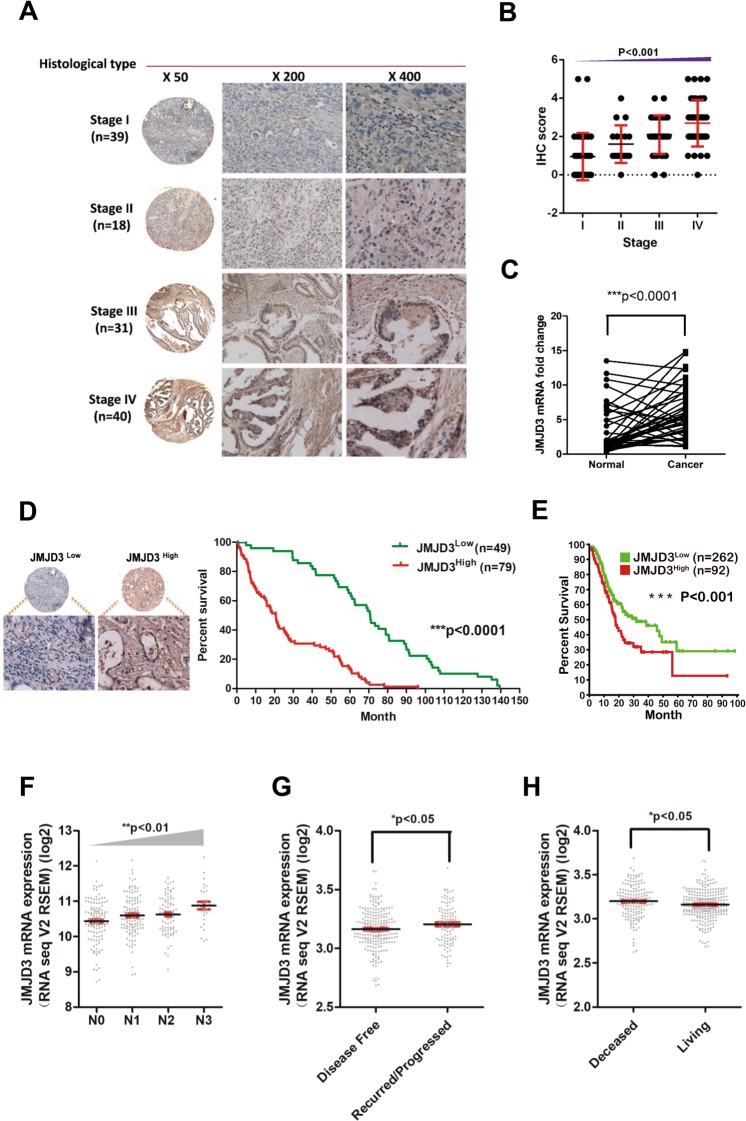
Expression level of JMJD3 in GC and its association with patient survival and clinical parameters. (**A**) Expression level of JMJD3 was determined by IHC staining in tissue microarray containing 128 GC patient samples. (**B**) Statistical analysis of IHC result in different tumor stage. (**C**) JMJD3 was upregulated in 41 GC patient cohort as determined by realtime PCR. (**D**) High JMJD3 expression predicts poor patient survival in the 128 GC patient cohort. (**E**) High JMJD3 expression predicts poor patient survival in TCGA dataset. (**F**) JMJD3 expression escalated with tumor stage as analyzed by cBioportal using TCGA data. (**G**) Disease free patient had lower level of JMJD3 compared with recurred/progressed patient as analyzed by cBioportal. (**H**) Living patient had lower level of JMJD3 compared with deceased patient as analyzed by cBioportal. *p < 0.05, **p < 0.01 and ***p < 0.001.

The expression level of JMJD3 was determined by IHC analysis in tissue microarray containing paraffin-embedded GC tissue samples from four different histological stages (Stage I n = 39; Stage II n = 18; Stage III n = 31; Stage IV n = 40). JMJD3 protein expression level significantly escalated (p < 0.001) with tumor stage (Fig. 1A,B). A total of 41 GC tissue and paired adjacent normal tissue samples were available from patients with GC who had undergone surgery. All patients were treated by radical gastrectomy and received no preoperative radiation or chemotherapy. Most patients (35/41) were at an early stage (stages 1 and 2) and no lymph node metastasis was present in any patient. The overall 5-year survival rate was 100%, suggesting that early diagnosis and surgical removal of the cancer tissue resulted in a good prognosis. Realtime PCR result of this cohort of patient sample demonstrated that JMJD3 level was significantly elevated in tumor compared with adjacent normal tissue (p < 0.001) (Fig. 1C). JMJD3 protein expression in gastric cancer specimens from 128 patients (Supplementary Table 1), including 89 male (69.5%) and 39 female (30.5%) (mean age was 59.3 years) was evaluated. According to JMJD3 expression level patients were categorized into two groups: low JMJD3 (n = 49) and high JMJD3 (n = 79) (Fig. 1D). The overall survival rate of patients with high JMJD3 expression was significantly lower compared to patients with low JMJD3 expression (p < 0.001) (Fig. 1D). We also analyzed patient survival according to JMJD3 expression level using TCGA data and obtained similar result (p < 0.001) (Fig. 1E). Furthermore, in the univariate Cox regression analysis, high JMJD3 expression was associated with a significantly increased risk of death (p < 0.001). Age (p < 0.05), tumor–node–metastasis (TNM) stage (p < 0.01) and lymph node metastasis (p < 0.001) were also significant predictors of overall survival (Table 1). After the adjustment for potential confounding factors, high JMJD3 expression (p < 0.01), age (p < 0.01), and TNM stage (p < 0.01) but not lymph node metastasis (p = 0.982) still predicted poorer survival in the multivariate model (Table 1). Moreover, we also evaluated the association of JMJD3 expression and clinical parameters by cBioportal. JMJD3 mRNA expression escalated with tumor stage (p < 0.01) and was higher in recurred/progressed disease than disease free (p < 0.05) and also higher in dead patients than living patients (p < 0.05) (Fig. 1F–H).

**Table 1:** Univariate and multivariate analysis of overall survival in GC patients (n = 128).

	Univariate analysis		Multivariate analysis	
Variable	RR (95% CI)	P value	RR (95% CI)	P value
Age, y	1.03 (1.00–1.05)	0.021	1.05 (1.02–1.0)	0.001
**Sex**				
M	1.55 (0.84–2.85)	0.158	1.48 (0.77–2.84)	0.236
F	1		1	
***H***. ***pylori*** **infection**				
Negative	1.45 (0.82–2.56)	0.201		
Positive	1			
**Lauren type**				
Intestinal	0.54 (0.22–1.35)	0.188		
Diffuse	1.33 (0.55–3.21)	0.532		
Mix	1			
**Stage**				
I	0.13 (0.06–0.29)	<0.001	0.11 (0.03–0.36)	<0.001
II	0.17 (0.05–0.55)	0.003	0.14 (0.04–0.50)	0.002
III	0.39 (0.20–0.75)	0.005	0.32 (0.16–0.63)	0.001
IV	1		1	
**Lymph node metastasis**				
No	0.24 (0.11–0.51)	<0.001	0.99 (0.30–3.22)	0.982
Yes	1		1	
**JMJD3**				
Low	0.27 (0.14–0.50)	<0.001	0.36 (0.18–0.70)	0.003
High	1		1	

### The underlying mechanism of JMJD3 dysregulation in GC

**Figure 2:**
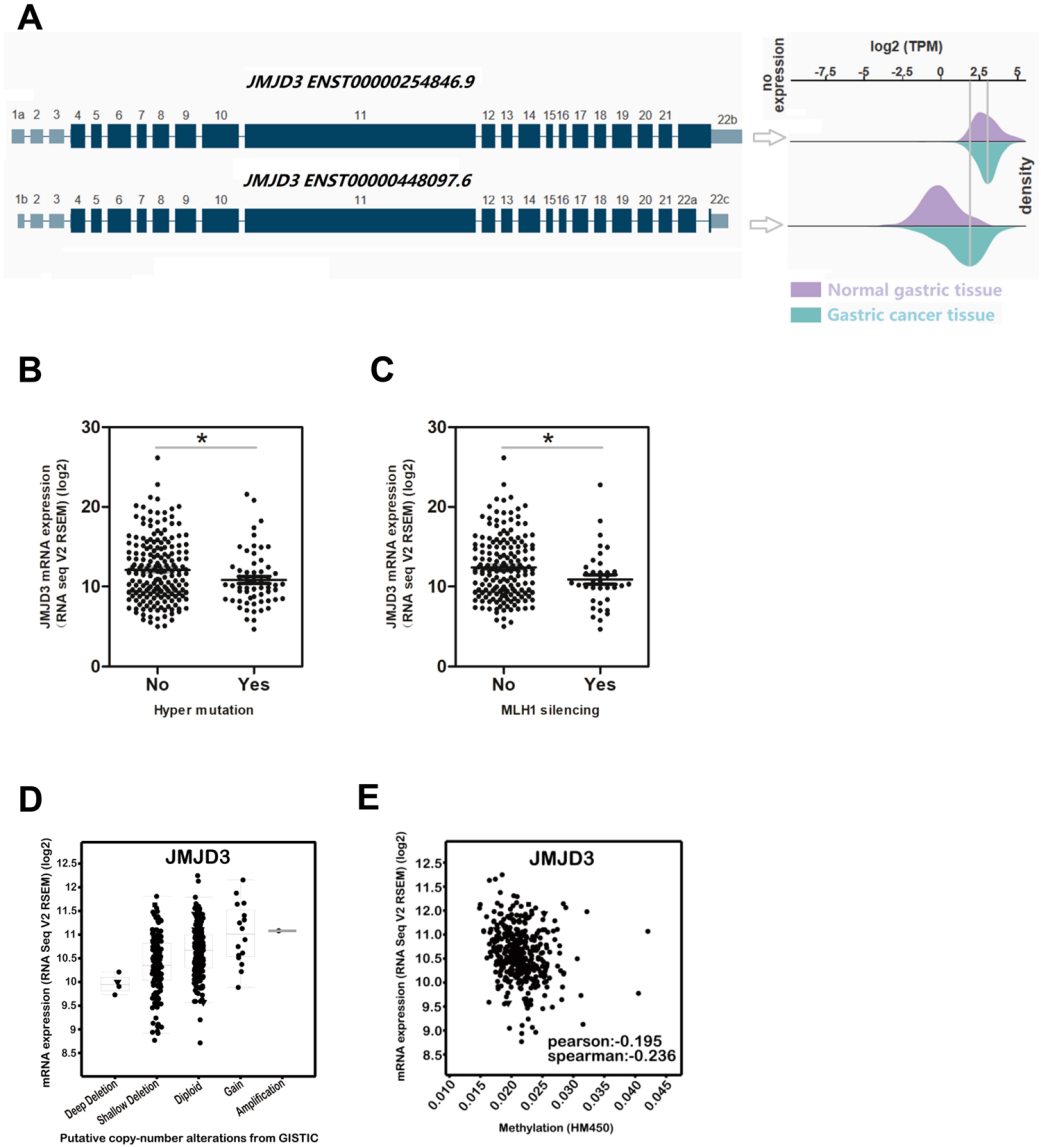
Deregulation of JMJD3 and its underlying mechanism by bioinformatics study. (**A**) The two transcript variants of JMJD3 were both upregulated in GC as analyzed by The Human Protein Atlas. (**B**) Patients with hypermutation had significantly lower level of JMJD3 expression as analyzed by cBioportal using TCGA data. (**C**) Patients with MLH1 silencing had significantly lower level of JMJD3 expression as analyzed by cBioportal using TCGA data. (**D**) Deletion, diploid, copy number gain and amplification were involved in the deregulation of JMJD3 expression as analyzed by cBioportal using TCGA data. (**E**) DNA methylation was also involved in JMJD3 deregulation stage as analyzed by cBioportal using TCGA data.*p < 0.05.

There are two transcript variants of JMJD3 from NCBI (Fig. 2A). Our result showed that the average expression level of both variants were higher in GC than in normal gastric tissue (Fig. 2A). Research evidence has shown that a proportion of colorectal cancer (CRC) are hypermutated, exhibit frequent epigenetic silencing of the MLH1 and have a better clinical prognosis^12^. Our result demonstrated that hyper mutation (Fig. 2B) and MLH1 silencing (Fig. 2C) were associated with lower JMJD3 expression (p < 0.05), supporting the oncogenic role of JMJD3. Genomic alteration analysis revealed that there were deep deletion, shallow deletion, diploid, copy number gain and amplification (Fig. 2D). JMJD3 mRNA expression was also negatively correlated with DNA methylation, although the correlation was weak (Fig. 2E).

### JMJD3 mutation and its influence on cell proliferation

**Figure 3:**
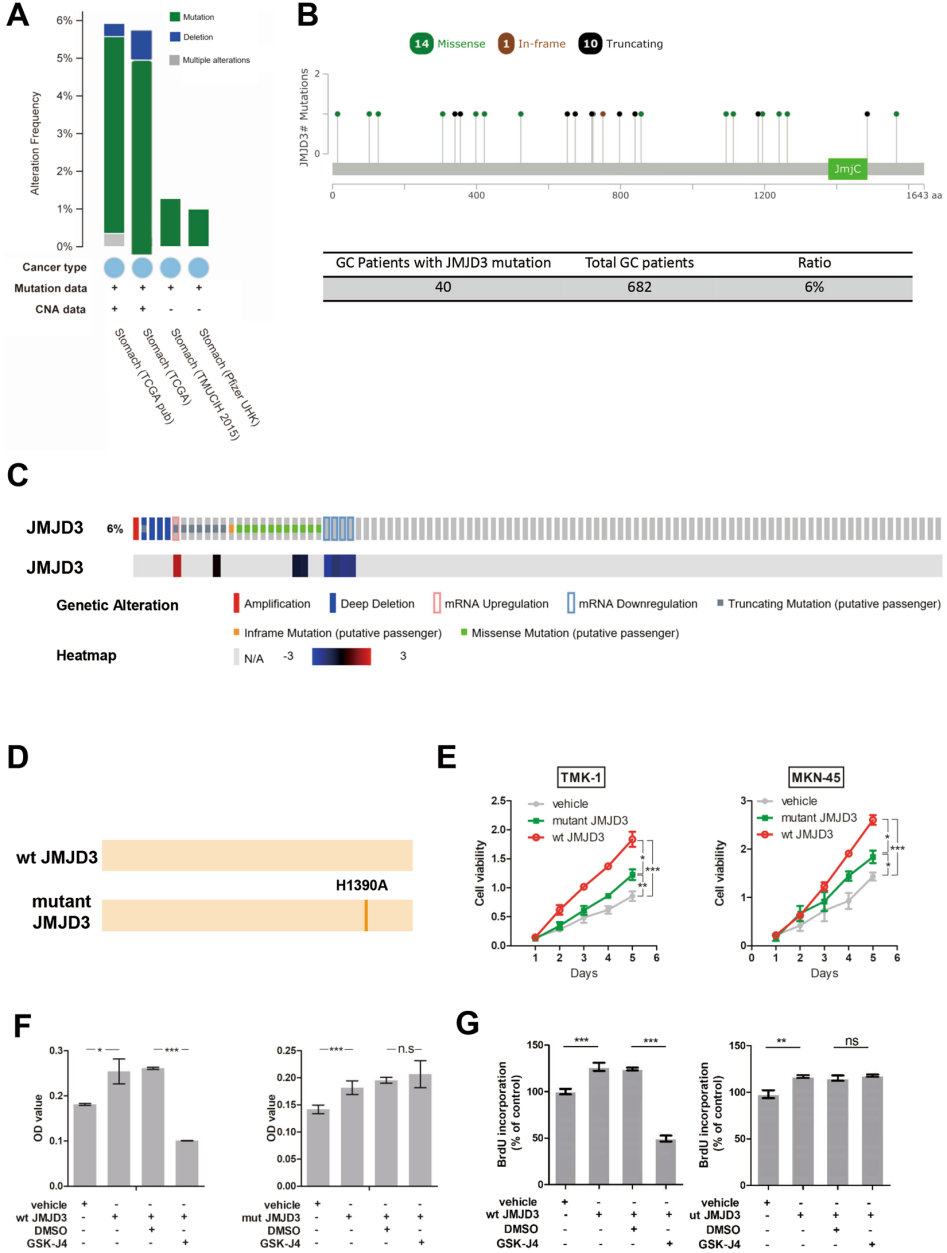
Mutation accounts for the majority of genetic aberration in JMJD3. (**A**) The percentage of JMJD3 genetic alteration in different GC patient cohorts analyzed by cBioportal. (**B**) The position of JMJD3 mutation in the four patient cohorts combined together cohorts analyzed by cBioportal. (**C**) Representative percentage of different genetic alteration in JMJD3 and the accompanying expression change cohorts analyzed by cBioportal. (**D**) Representative diagram of the two pMSCV plasmids established for functional study. (**E**) MTT result after transfection of wildtype or mutant JMJD3 plasmids in two GC cell lines. (**F**) MTT result after transfection of JMJD3 plasmids with or without GSK-J4. (**G**) BrdU result after transfection of JMJD3 plasmids with or without GSK-J4. *p < 0.05, **p < 0.01 and ***p < 0.001.

We analyzed JMJD3 genetic aberration and found mutation accounts for the majority of genetic alteration of JMJD3 (Fig. 3A). Further analysis revealed that in a cohort of 682 GC patients, approximately 6% patients have JMJD3 mutation including missense mutation, in-frame mutation and truncating mutation. Mutation distributed in areas other than the JmjC domain (Fig. 3B), which is a catalytic domain required for the demethylase activity of JMJD3. Representative proportion for each type of genetic alteration including amplification, deletion, mRNA upregulation and downregulation and mutation in JMJD3 was shown in Fig. 3C. JMJD3 expression level was changed accompanying these alterations (Fig. 3C). To confirm the function of JMJD3 mutation we generated two pMSCV plasmids expressing wildtype JMJD3 (wt JMJD3) or mutant JMJD3 which has lost the histone demethylase function (Fig. 3D). MTT result revealed that overexpression of wildtype and mutant JMJD3 significantly promoted cell viability in two GC cell lines. However, the promoting effect for mutant JMJD3 was significantly weaker than that of wildtype JMJD3 (Fig. 3E), suggesting that there was other mechanism involved in the oncogenic function of JMJD3 other than its histone demethylation function. To further confirm the result, we used a JMJD3 histone demethylation inhibitor GSK-J4 for MTT, BrdU and transwell experiments. Result showed that the proliferation promoting effect of wildtype JMJD3 could be partially reversed by GSK-J4 while GSK-J4 could not reverse the promoting effect of mutant JMJD3 (Fig. 3F,G). Similar result was found for cell migration (Supplementary Fig. 1). These results suggest that both demethylase-dependent and demethylase-independent mechanisms were involved in the oncogenic function of JMJD3.

### Potential targets of JMJD3 in GC

**Figure 4:**
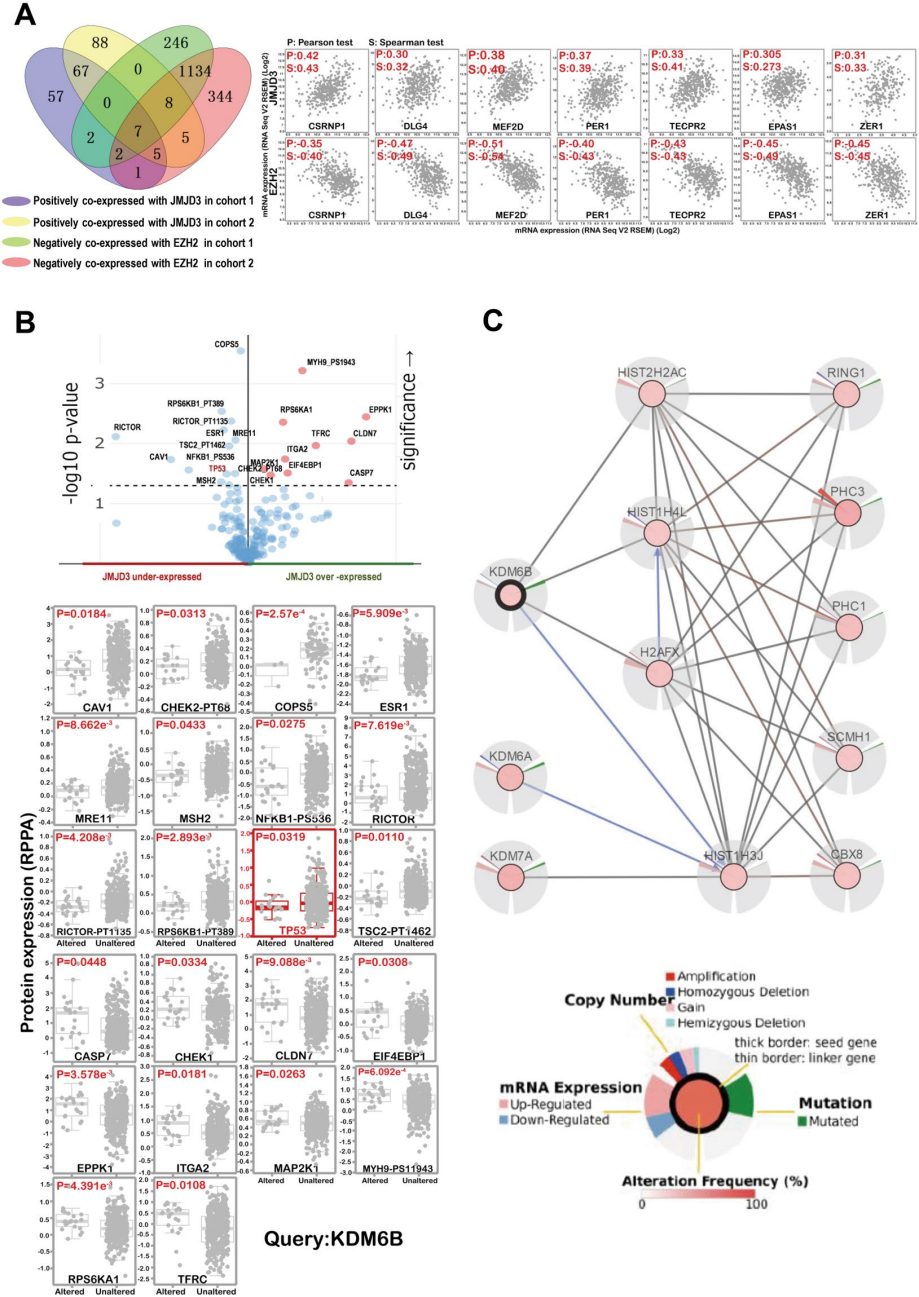
The downstream targets of JMJD3 by bioinformatics study. (**A**) Genes that were positively co-expressed with JMJD3 and at the same time negatively co-expressed with EZH2 were analyzed by Venny using TCGA data. (**B**) Genes that were significantly influenced by JMJD3 expression alteration were analyzed by cBioportal. (**C**) Net work analysis was performed in cBioportal and four direct and five indirect targets of JMJD3 were found.

To identify proteins that are potentially regulated by JMJD3, we performed different analysis. H3K27me3 levels are determined by the balance between activities of histone methyltransferase EZH2 and histone demethylase JMJD3^9^. We analyzed proteins that are positively co-expressed with JMJD3 (2 cohorts) and at the same time negatively co-expressed with EZH2 (2 cohorts) and found 7 potential targets (Fig. 4A). When validating these potential targets in patients samples, we found that three targets named CSRNP1, MEF2D and EPAS1 are significantly up-regulated in tumor tissues when compared with normal tissues (Supplementary Fig. 1). This is consistent with the previous result that they are positively co-expressed with JMJD3 which is overexpressed in GC. We also analyzed proteins that are significantly upregulated accompanying JMJD3 overexpression or downregulated accompanying JMJD3 underexpression. Twenty two potential targets were discovered for JMJD3 by this means including TP53, which has already been reported to be JMJD3 target^13^ (Fig. 4B). Furthermore, we investigated interacting proteins for JMJD3 using cBioportal network analysis. We identified 4 proteins that are directly regulated by JMJD3 and 5 proteins that are indirectly affected by JMJD3 (Fig. 4C).

## Discussion

Gastric cancer (GC) is one of the leading causes of cancer deaths worldwide and the incidence and mortality rates are higher in Asian compared with Western countries^2^. In China, GC is the second most prevalent type of cancer, with 679,100 new cases and 498,000 deaths reported in 2015^14^. Thus, investigating promising diagnostic and/or prognostic markers for GC is urgent and vitally important. Recently, JMJD3 has been highlighted in a variety of studies of human malignancies due to the increasing focus on histone methylation in cancer development^15^. To the best of our knowledge, this is the first study to determine the dysregulation of JMJD3 in GC and to evaluate the prognostic implications of overexpressed JMJD3 protein.

Result of IHC analysis of 128 gastric cancer tissues from four different histological stages indicated that JMJD3 protein expression is increasingly elevated with histological stage (Fig. 1A,B). Moreover, this study showed that JMJD3 transcripts were significantly increased in gastric cancer tissues from 41 cases when compared with paired adjacent normal tissues (Fig. 1C). We also found that high JMJD3 expression predicted unfavorable survival both in the 128 patients cohort (Fig. 1D) and in TCGA data (Fig. 1E). Multivariate analysis of the 128 patient cohort showed that JMJD3 was an independent prognosis predictor in GC (Table 1). Consistent with our result, bioinformatics analysis in cBioportal also revealed that JMJD3 expression increased along with tumor stage (Fig. 1F) and high expression of JMJD3 was associated with poor disease progression (Fig. 1G,H). The two JMJD3 transcript variants were also found to be upregulated in GC compared with normal tissue from The Human Protein Atlas (Fig. 2A). In accordance with our findings, Shen *et al*. observed that JMJD3 transcripts were significantly elevated in renal carcinoma and that JMJD3 expression was higher in cancer tissue compared to adjacent normal tissue^8^. JMJD3 expression was also found to be significantly higher in prostate cancer and further increased during metastasis^9,10^. Similarly, Sui *et al*. reported that JMJD3 expression was upregulated in glioma^11^. Conversely, Agger *et al*. found a significant decrease in JMJD3 expression in various cancers, including lung and liver carcinomas, as well as various hematopoietic malignancies^6^. Moreover, JMJD3 expression levels are lower in colorectal cancer relative to normal tissues^7^. Based on the above reports, we conclude that the expression patterns of JMJD3 are context and cancer type specific. For example, JMJD3 can promote breast cancer cell invasion through upregulation of the EMT-specific gene-SNAI1^16^. However, in colorectal cancer cells, a decrease in JMJD3 can significantly increase cell proliferation through cell cycle progression and apoptosis suppression^7^.

Previous study has shown that a better clinical prognosis was found in some CRC patients in which there were hypermutation and epigenetic silencing of the MLH1^12^. We analyzed JMJD3 expression, mutation status and MLH1 silencing in GC and our result demonstrated that hyper mutation (Fig. 2B) and MLH1 silencing (Fig. 2C) were associated with lower JMJD3 expression. Hyper mutation and MLH1 silencing were also found to be associated with longer overall survival in GC^17,18^. This is consistent with our finding that lower JMJD3 level predicts better survival in GC.

Our bioinformatics study also demonstrated that both genetic alteration and DNA methylation might participate in the dysregulation of JMJD3 in GC (Fig. 2D,E). Up to now, there has been few reports about mutation of JMJD3 in cancer. We further comprehensively investigated JMJD3 mutation in cBioportal (Fig. 3A–C). Results showed that in a total of 682 GC patients around 6% patients have JMJD3 mutation and the mutation was not in the catalytic domain. The functional impact of JMJD3 mutation (loss of demethylase function) was also studied by overexpressing mutant JMJD3 plasmids or using pharmacological inhibitor (Fig. 3D–G and Supplementary Fig. 1) and we found that JMJD3 promotes GC cell proliferation and migration by both demethylase-dependent and demethylase-independent mechanisms. The demethylase-independent function of JMJD3 has been reported but not in cancer^19,20^. The exact mechanism underlying the oncogenic function of JMJD3 needs further elucidation.

We also investigated the potential targets of JMJD3 by different bioinformatics methods. Totally 29 proteins were found significantly affected by JMJD3 and another 4 targets were found directly regulated by JMJD3 by network analysis (Fig. 4A–C). We validated the expression of potential targets that are positively co-expressed with JMJD3 and negatively co-expressed with EZH2 by realtime PCR. Results showed that CSRNP1, MEF2D and EPAS1 are significantly up-regulated in tumor tissues compared with normal tissues (Supplementary Fig. 2). Among them, the oncogenic role of MEF2D, EPAS1 has been well established^21,22^. MEF2D was upregulated in GC and was significantly associated with the clinical parameters. Its high expression predicts shorter overall survival^23^. EPAS1 also called hypoxia-inducible factor-2α (HIF-2α), has been reported to play an oncogenic role in various cancers including gastric cancer, colon cancer, lung cancer, pancreatic cancer, renal cancer, etc^24–28^. It is also a promising target for cancer therapy^29,30^. In contrary to our finding, CSRNP1 (AXUD1) has been reported to be decreased in cancers^31^ and play a pro-apoptotic role^32^. Besides, we also discovered twenty two potential targets that showed similar trend of expression change with JMJD3 in GC (Fig. 4B). The role of CAV1 in gastric cancer remains controversial^33^. The oncogenic role of COPS5^34^, MRE11^35^, RICTOR^36^, CHEK1^37^, CLDN7^38^, EIF4EBP1^39^, EPPK1^40^, ITGA2^41^, MAP2K1^42^, RPS6KA1^43^, TFRC^44^ have been reported in different cancer types. Specifically EIF4EBP1 were overexpressed in GC^45^ and MAP2K1 has been identified to be associated with GC by different studies^46^. Furthermore, high expression of MRE11 complex^47^, RICTOR^48^, ITGA2^49^, MAP2K1^46^ predicts poor survival in GC. ITGA2 has been reported to be a target of EZH2^50^, suggesting it is very probably a JMJD3 target. Among the four genes that were found directly regulated by JMJD3 by network analysis (Fig. 4C), HIST2H2AC was reported increased in breast cancer^51^ and HIST1H4L was increased in prostate cancer^52^. Altogether, our findings demonstrated that it is highly possible that some of these potential targets are regulated by JMJD3 in GC. However, the direct interaction of these novel targets with JMJD3 awaits further validation.

In summary, our study report for the first time the oncogenic role of JMJD3 in GC and inhibition of JMJD3 might be an effective therapeutic approach for GC. Some inhibitors that suppress the histone demethylase activity of JMJD3 have been identified, such as JIB-04 and GSK-J1/4. They have been reported to exert tumor suppressive activity in several malignances, including breast cancer^53^, osteoblast cancer^54^, leukemia^55^, ovarian cancer stem cells^56^ and pediatric brainstem glioma^57^. These inhibitors may have therapeutic potential for GC via targeting JMJD3. Although the functional role of JMJD3 in GC is unclear, it is an emerging therapeutic epigenetic target for cancer treatment and might be associated with the progression of GC and serves as a prognostic indicator.

## Supplementary information

Supplementary Dataset 1
