# Supplementary material for: Comprehensive profiling of JMJD3 in gastric cancer and its influence on patient survival
Source: Sci Rep. 2019 Jan 29;9:868. doi: 10.1038/s41598-018-37340-w (PMC6351656; doi:10.1038/s41598-018-37340-w)
Supplement: Supplementary file 1 — Supplementary Dataset 1 [file 41598_2018_37340_MOESM1_ESM.pdf]

Comprehensive profiling of JMJD3 in gastric cancer and its influence on patient survival

Zhenyu Xu[1#], Yabin Xia[2#], Zhangang Xiao[3#], Yuliang Jia[4], Lina Li [5], Yan Jin[2], Qijie Zhao[3], Lin Wan[6], Tao Yi[7], Yangyang Yu[8], Qinglian Wen[9], Yinxin Zhu[10], Bo Qin[11], Fan Zhang [12*], Jing Shen[3*]

Supplementary Table 1. Demographics of 128 GC patients and tumor variable analysis

Variable	Alive	%	Dead	%	P value
	(n=74)		(n= 54)		
**Age, mean ±SD**	59.3±12.9		63.1±11.13		0.081
**Sex**					
**M**	49	55.1	40	44.9	0.34
**F**	25	64.1	14	35.9	
**H. pylori infection**					
**Negative**	40	53.3	35	46.7	0.176
**Positive**	34	65.4	18	34.6	
**Lauren type**					
**Intestinal**	41	68.3	19	31.7	0.075
**Diffuse**	25	47.2	28	52.8	
**Mix**	8	57.1	6	42.9	
**Stage**					
**I**	32	80	8	20	<.001
**II**	10	76.9	3	23.1	
**III**	20	60.6	13	39.4	
**IV**	12	29.3	29	70.7	
**Lymph node metastasis**					
**No**	31	79.5	8	20.5	0.001
**Yes**	43	48.9	45	51.1	
**JMJD3**					
**Low**	34	69.4	15	30.6	0.037
**High**	40	50.6	39	49.4	

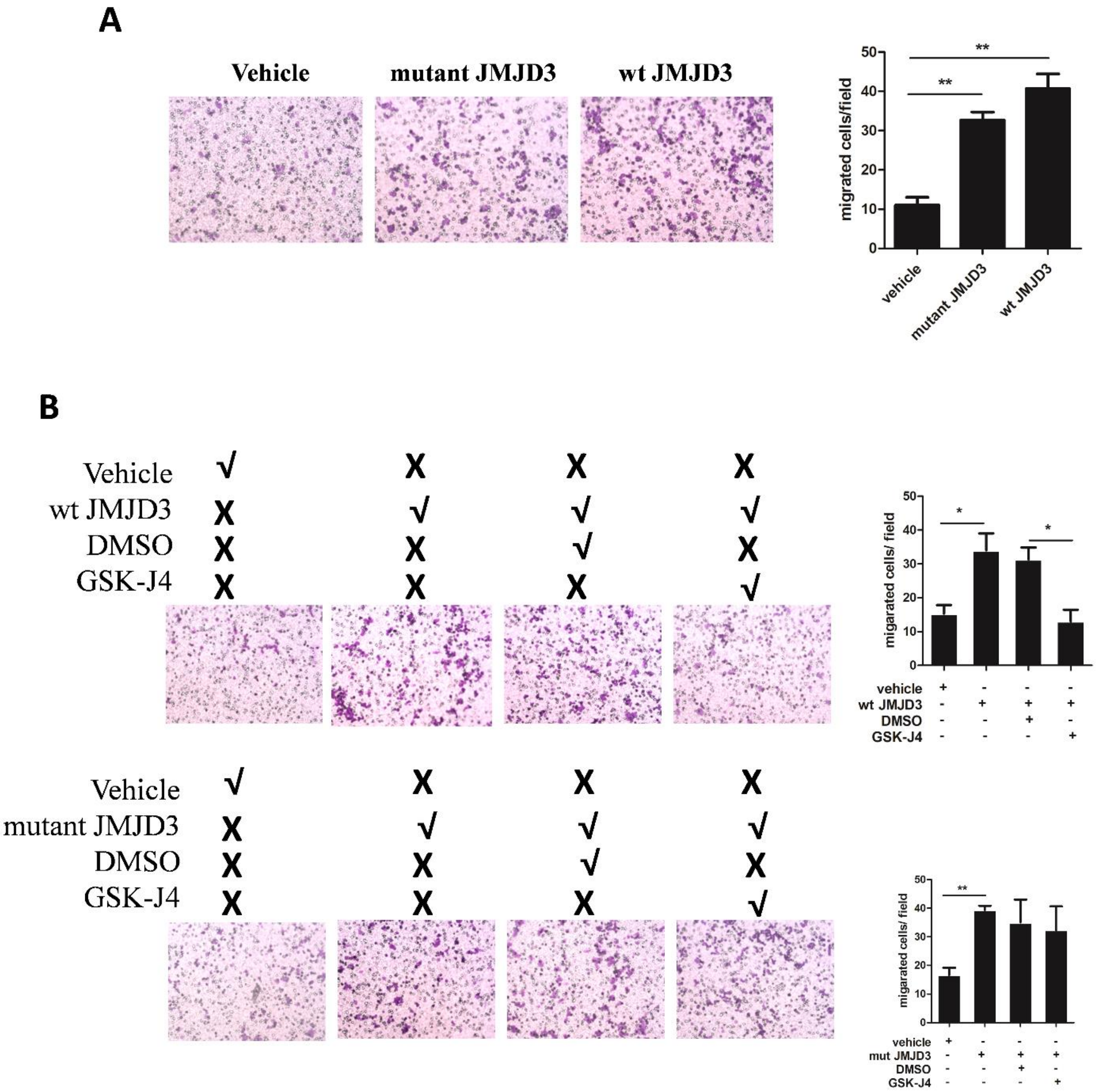

Supplementary figure 1. JMJD3 inhibit GC cell migration in in vitro studies. A Transwell result after transfection of wildtype or mutant JMJD3 plasmids in two GC cell lines. B. Transwell result after transfection of JMJD3 plasmids with or without GSK-J4. * $p < 0.05$, ** $p < 0.01$.

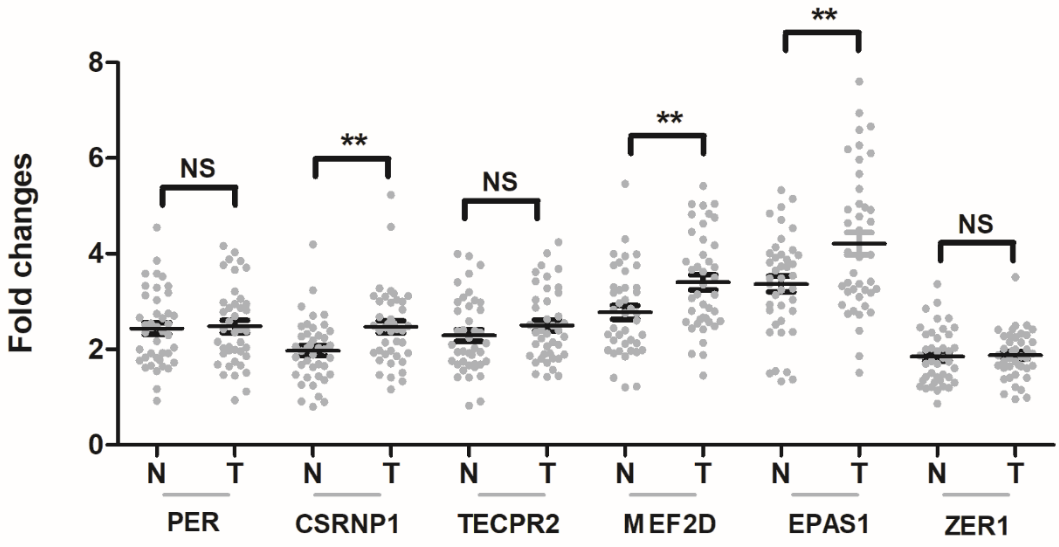

Supplementary figure 2. The expression of JMJD3 potential targets are measured in GC patient samples. The expressiom of JMJD3 potential targets including PER, CSRNP1, TECPR2, MEF2D, EPAS1, ZER1 was measured by RT-qPCR in GC tumor and normal tissues. ** $p < 0.01$. Primers used are as follows: PER, Forward Sequence: 5’-TCAACTGCCTGGACAGCATCCT-3’; Reverse Sequence: 5’-TCAGAGGCTGAGGAGGTGGTAT-3’; CSRNP-1, Forward Sequence: 5’-GCGCTTGAAAGAGGAGAAGTTGG-3’; Reverse Sequence: 5’-CCACAGCGACTGCCAAGTCCT-3’; TECPR2, Forward Sequence: 5’-AGGAGATGACGACCATTGGTGG-3’; Reverse Sequence: 5’-GCGCAGCTTATCTGCCACCTTT-3’; MEF2D, Forward Sequence: 5’-AGGGAAATAACCAAAAAACTACCAAA-3’; Reverse Sequence: 5’-GCTACATGAACACAAAAACAGAGACC-3’; EPAS1, Forward Sequence: 5’-CTGTGTCTGAGAAGAGTAACTTCC-3’; Reverse Sequence: 5’-TCTGCCAGGTAGACAACGACCA-3’; ZER1, Forward Sequence: 5’-TCTGCCAGGTAGACAACGACCA-3’; Reverse Sequence: 5’-AGGAGAACTCCATGACCTGGTC-3’.
